# Complete Chloroplast Genome Sequence of Poisonous and Medicinal Plant *Datura stramonium*: Organizations and Implications for Genetic Engineering

**DOI:** 10.1371/journal.pone.0110656

**Published:** 2014-11-03

**Authors:** Yang Yang, Dang Yuanye, Li Qing, Lu Jinjian, Li Xiwen, Wang Yitao

**Affiliations:** 1 State Key Laboratory of Quality Research in Chinese Medicine, Institute of Chinese Medical Sciences, University of Macau, Macau, China; 2 Department of Pharmacy, Shanghai Changzheng Hospital, Second Military Medical University, Shanghai, China; 3 Institute of Chinese Materia Medica, China Academy of Chinese Medical Sciences, Beijing, China; University of Western Sydney, Australia

## Abstract

*Datura stramonium* is a widely used poisonous plant with great medicinal and economic value. Its chloroplast (cp) genome is 155,871 bp in length with a typical quadripartite structure of the large (LSC, 86,302 bp) and small (SSC, 18,367 bp) single-copy regions, separated by a pair of inverted repeats (IRs, 25,601 bp). The genome contains 113 unique genes, including 80 protein-coding genes, 29 tRNAs and four rRNAs. A total of 11 forward, 9 palindromic and 13 tandem repeats were detected in the *D. stramonium* cp genome. Most simple sequence repeats (SSR) are AT-rich and are less abundant in coding regions than in non-coding regions. Both SSRs and GC content were unevenly distributed in the entire cp genome. All preferred synonymous codons were found to use A/T ending codons. The difference in GC contents of entire genomes and of the three-codon positions suggests that the *D. stramonium* cp genome might possess different genomic organization, in part due to different mutational pressures. The five most divergent coding regions and four non-coding regions (*trnH-psbA*, *rps4-trnS*, *ndhD-ccsA*, and *ndhI-ndhG*) were identified using whole plastome alignment, which can be used to develop molecular markers for phylogenetics and barcoding studies within the Solanaceae. Phylogenetic analysis based on 68 protein-coding genes supported *Datura* as a sister to *Solanum*. This study provides valuable information for phylogenetic and cp genetic engineering studies of this poisonous and medicinal plant.

## Introduction

Scopolamine is an important tropane alkaloid from Solanaceae plants widely used as anticholinergic agent that acts on the parasympathetic nervous system [1]. It is widely used as sedative in clinical practice including preanesthetic medication for general anesthesia, and also for manic psychosis, motion sickness, parkinsonism and organophosphorus pesticide poisoning [2], [3]. Due to its activity in exciting the respiratory center and sedative effect on the cerebral cortex, scopolamine is used to rescue respiratory failure caused by extremely heavy epidemic encephalitis, accompanied by severe frequent tics in such condition [4], [5]. Recently scopolamine also exhibited great potential as a drug for use in withdrawal for heroin addicts [6]. Scopolamine occurres in all plant organs and was traditionally extracted from flowers of *Datura* species. It was recently reported that the maximum concentrations were found in the stems and leaves of juvenile plants [3], [7]. But the concentration is still quite low and its supply cannot meet the market demand. Therefore significant attention has been paid to its commercial production using biotechnologies.

Over the last decades, engineering techniques have been intensively investigated as a possible tool for the production of scopolamine in different plant species that produce tropane alkaloids,including overexpression of genes involved in the biosynthesis of scopolamine [1], [8], [9] as well as biotransforming hyoscyamine into scopolamine in hairy root cultures [9]–[11]. However production was too low for commercialization. Because of the complicated metabolic pathway of biosynthesis, it has become clear that unorganized plant tissue cultures are frequently unable to produce scopolamine at the same levels as the intact plant [8].

Plastids of higher plant are cellular organelles with circular genomes of 120–160 kb in size present in 1,000–10,000 copies per cell [12], and are maternally inherited in most angiosperm plant species [13]. Chloroplast transformation offers a higher level expression of foreign genes in intact plant compared with hair root cultures. In the past two decades, more than forty transgenes have been stably integrated and expressed in the tobacco cp genome to confer important agronomic traits or produce commercial products including biomaterials and recombinant proteins [8]. Chloroplast engineering, either alone or in combination with traditional cultivation techniques, may provide the means to develop novel sources of plants to solve tropane alkaloid biosynthesis, the century old problem. Great progress has been made in the study of discovering rate-limiting enzymes in the key steps of catalysis for tropane alkaloids synthesis [1], [10].

However the lack of plastid genome data available in public databases limits further studies of cp transformation. *Datura stramonium* has been one of the major plant sources for extracting scopolamine. It is a good model plant to study at the biochemical and molecular level. We here analyzed and characterized the cp genome of *D. stramonium*, providing the basic genetic information for cp engineering. Comparison of the genome structures with other plant species was also determined. These data should also contribute to a better understanding in future studies of evolution within the asteridae clade and species identification of this poisonous and medicinal plant.

## Materials and Methods

### Genome Sequencing Preparation

Chloroplast DNA (cp DNA) was extracted from approximately 100 g fresh young leaves of *Datura stramonium* using a sucrose gradient centrifugation method that was improved by Li *et al.* [14]. The concentration of the DNA for each cp genome was estimated by measuring A260 with an ND-2000 spectrometer (Nanodrop technologies, Wilmington, DE, USA), and visual approximation was performed using gel electrophoresis. Pure cpDNA was sequenced using a 454/Roche FLX high-throughput sequencing platform.

### Genome Assembly and Annotation

The Sff-file obtained was pre-processed, including the trimming of low-quality sequences. *De novo* assembly was performed using version 2.5 of the GS FLX system software. The position and direction of the contigs were identified using the cp genome sequence of *Nicotiana sylvestris* (NC_007500) as the reference sequence. The boundaries of IR-LSC and IR-SSC were confirmed using PCR amplification. We used the online program DOGMA (Dual Organellar GenoMe Annotator) [15] to annotate the cp genome. The position of each gene was determined using a blast method with the complete cp genome sequence of *N. sylvestris* as a reference sequence. Minor revisions were performed according to the start and stop codons. The tRNA genes were identified using DOGMA and tRNAscan-SE [16]. The nomenclature of cp genes followed the ChloroplastDB [17]. The circular cp genome map was drawn by the OGDRAW program [18]. To analyze the characteristics of variations in synonymous codon usage by neglecting the influence of amino acid composition, the relative synonymous codon usage values (RSCU) were determined using MEGA5.2 [19]. The final cp genome of *Datura stramonium* has been deposited to GenBank (accession number NC_018117).

### Genome Comparison and Sequence Analysis

The pairwise alignments of cp genomes were performed using MUMmer [20]. The mVISTA program in Shuffle-LAGAN mode [21] was used to compare the cp genome of *Datura stramonium* with three other cp genomes using the genome sequence of *Datura stramonium* as reference. We used DnaSP v5 [22] to calculate the substitution rates. Simple sequence repeats (SSRs) were detected using MISA (http://pgrc.ipkgatersleben.de/misa/), with thresholds of eight repeat units for mononucleotide SSRs, four repeat units for di- and trinucleotide SSRs and three repeat units for tetra-, penta- and hexanucleotide SSRs. All of the repeats found were manually verified, and the redundant results were removed. We investigated the distribution of SSRs located in LSC, SSC and IR regions. The proportions of different nucleotides (A, T, C, G) were calculated and different chloroplast SSR types (CSTs) found among SSRs were discovered. To determine the repeat structure, REPuter [23] was used to visualize both forward and palindrome repeats. The settings for the minimal repeat size was 30 bp and the identity of repeats was no less than 90% (hamming distance  = 3). Low complexity and nested repeats were ignored. Tandem repeats were analyzed with the aid of Tandem Repeats Finder (TRF) v4.04 [24] and the parameters were set according to Nie *et al* [25].

### Phylogenetic Analysis

In order to identify the phylogenetic position of *Datura* within the asterid lineages, 42 complete cp genome sequences are downloaded from the Genbank of NCBI database (Table S1). Protein-coding gene sequences (Table S2) were aligned using the ClustalW2 algorithm [26]. Pairwise sequence divergences were calculated using Kimura two-parameter (K2P) model [27]. And 68 protein-coding genes (Table S2) shared by all studied plastid genomes were extracted for phylogenetic analysis. Each gene was aligned using the ClustalW and the alignment was edited manually. Maximum likelihood (ML) analysis was performed using RAxML v7.0 [28] using the GTR+I+G nucleotide substitution model under the best fit parameters determined by Modeltest ver. 3.7 [29]. Maximum Parsimony (MP) analysis was performed using PAUP ver. 4.0b10 [30] taking the cp genome sequence of *Cycas taitungensis* (NC_009618) as the outgroup. MP searches included 1,000 replicates of random taxon addition and a heuristic search using tree bisection and reconnection (TBR) branch swapping (Multrees option in effect). Both of these analyses, we using 1000 bootstrap replicates.

## Result

### Genome Features

**Figure 1:**
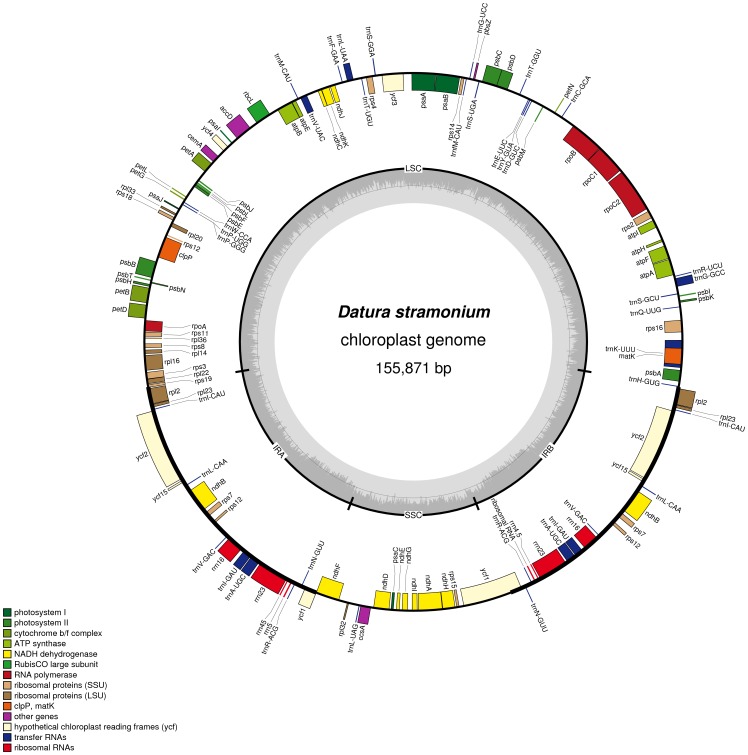
Gene map of the *Datura stramonium* chloroplast genome. Genes drawn inside the circle are transcribed clockwise, and those outside are counterclockwise. Genes belonging to different functional groups are color-coded. The darker gray in the inner circle corresponds to GC content, while the lighter gray corresponds to AT content.

The complete cpDNA genome of *Datura stramonium* is 155,871 bp in length (GeneBank: NC_018117) with a typical quadripartite structure of land plant cp genomes. The cp genome are divided into a LSC (86,302 bp) and a SSC (18,367 bp) regions separated by a pair of inverted repeat regions (IRa and IRb) of 25,602 bp (Table 1, Figure 1). The overall GC content of the whole cp genome sequence is 37.9% which is similar to those of the other reported asteridae cp genomes [31]–[35]. However the GC content is unevenly distributed in the entire cp genome. It is highest in the IR regions (43.1%), median in the LSC region (36.0%) and lowest in the SSC regions (32.3%).

**Table 1:** Base composition in the *Datura stramonium* chloroplast genome.

	T (U) (%)	C (%)	A (%)	G (%)	Length (bp)
LSC	32.7	18.4	31.3	17.6	86,302
SSC	34.0	16.8	33.7	15.5	18,367
IRa	28.3	20.7	28.6	22.4	25,601
IRb	28.6	22.4	28.3	20.7	25,601
Total	31.5	19.2	30.6	18.6	155,871
CDS	31.2	17.9	30.6	20.3	80,316
1st position	23.7	18.9	30.6	26.8	26,772
2nd position	32.5	20.3	29.3	17.9	26,772
3rd position	37.6	14.3	31.8	16.3	26,772

The positions of all the genes identified in the *D. stramonium* cp genome and functional categorization of these genes are presented in Figure 1. This cp genome encodes 132 predicted functional genes, of which 113 genes are unique, including 80 protein-coding genes, 29 transfer RNA (tRNA) genes and four rRNAs (Table S3). In addition, seven tRNA, all rRNA and eight protein-coding genes are duplicated in the IR regions. The LSC region contains 62 protein-coding and 25 tRNA genes but one tRNA and 11 protein-coding genes in the SSC region. There are altogether 14 intron-containing genes, 11 (nine protein-coding and two tRNA genes) of which contain one intron and three (*rps12*, *clpP* and *ycf3*) of which contain two introns (Table S4). The *rps12* gene is trans-spliced and the 5′ end located in the LSC region and the two duplicated 3′ end are in the IR regions. The *ndhA* gene has the longest intron (1,155 bp).

Protein-coding regions accounted for 59.7% of the whole genome sequence, while rRNA and tRNA regions accounted for 4.5% and 5.8%, respectively. The remaining regions are non-coding sequences, including introns, intergenic spacers and pseudogenes. Moreover, the total length of all the 88 protein-coding genes is 80,316 bp and these genes comprise 26,772 codons. Frequency of codon usage was calculated in the *D. stramonium* cp genome, and summarized in Table 2. A total of 10.6% of all codons (2,848) encodes leucine, and 1.1% of which (305) encodes cysteine, which are the most and least prevalent amino acids, respectively. Within protein-coding sequences (CDS), the percentage of AT content of the first, second and third codon positions are 54.3%, 61.8% and 69.4%, respectively (Table 1). Such bias towards a higher AT representation at the third codon position was also observed in other land plant cp genomes [25], [31], [36], [37]. There were 96.7% (29/30) of all the types of preferred synonymous codons (RSCU>1) ending with A or U and 90.6% (29/32) of non-preferred synonymous codons (RSCU <1) ending with G or C. In addition, A- and/or U-ending codons account for 69.3% of all codons within CDS. The usage of start codon (AUG) and UGG coding trp has no bias (RSCU = 1).

**Table 2:** The codon–anticodon recognition pattern and relative synonymous codon usage (RSCU) for the *Datura stramonium* chloroplast genome.

Amino acid	Codon	Count	RSCU	tRNA
Phe	UUU	955	1.27	
Phe	UUC	551	0.73	trnF-GAA
Leu	UUA	875	1.84	trnL-UAA
Leu	UUG	579	1.22	trnL-CAA
Leu	CUU	612	1.29	
Leu	CUC	207	0.44	
Leu	CUA	378	0.80	trnL-UAG
Leu	CUG	197	0.42	
Ile	AUU	1105	1.47	
Ile	AUC	461	0.61	trnI-GAU
Ile	AUA	684	0.91	trnI-CAU
Met	AUG	622	1.00	trn(f)M-CAU
Val	GUU	526	1.46	
Val	GUC	182	0.50	trnV-GAC
Val	GUA	539	1.46	trnV-UAC
Val	GUG	197	0.55	
Ser	UCU	591	1.70	
Ser	UCC	341	0.98	trnS-GGA
Ser	UCA	407	1.17	trnS-UGA
Ser	UCG	210	0.60	
Pro	CCU	428	1.52	
Pro	CCC	206	0.73	trnP-GGG
Pro	CCA	334	1.18	trnP-UGG
Pro	CCG	162	0.57	
Thr	ACU	540	1.58	
Thr	ACC	267	0.78	trnT-GGU
Thr	ACA	410	1.20	trnT-UGU
Thr	ACG	148	0.43	
Ala	GCU	616	1.75	
Ala	GCC	245	0.70	
Ala	GCA	400	1.14	trnA-UGC
Ala	GCG	143	0.41	
Tyr	UAU	783	1.60	
Tyr	UAC	198	0.40	trnY-GUA
Stop	UAA	44	1.52	
Stop	UAG	25	0.86	
His	CAU	482	1.53	
His	CAC	147	0.47	trnH-GUG
Gln	CAA	705	1.49	trnQ-UUG
Gln	CAG	244	0.51	
Asn	AAU	1003	1.52	
Asn	AAC	317	0.48	trnN-GUU
Lys	AAA	1052	1.48	trnK-UUU
Lys	AAG	371	0.52	
Asp	GAU	860	1.60	
Asp	GAC	217	0.40	trnD-GUC
Glu	GAA	1036	1.47	trnE-UUC
Glu	GAG	370	0.53	
Cys	UGU	223	1.46	
Cys	UGC	82	0.54	trnC-GCA
Stop	UGA	18	0.62	
Trp	UGG	475	1.00	trnW-CCA
Arg	CGU	341	1.26	trnR-ACG
Arg	CGC	100	0.37	
Arg	CGA	394	1.46	
Arg	CGG	126	0.47	
Arg	AGA	487	1.80	trnR-UCU
Arg	AGG	174	0.64	
Ser	AGU	420	1.21	
Ser	AGC	119	0.34	trnS-GCU
Gly	GGU	578	1.26	
Gly	GGC	199	0.43	trnG-GCC
Gly	GGA	740	1.61	trnG-UCC
Gly	GGG	324	0.70	

### SSR Analysis

**Figure 2:**
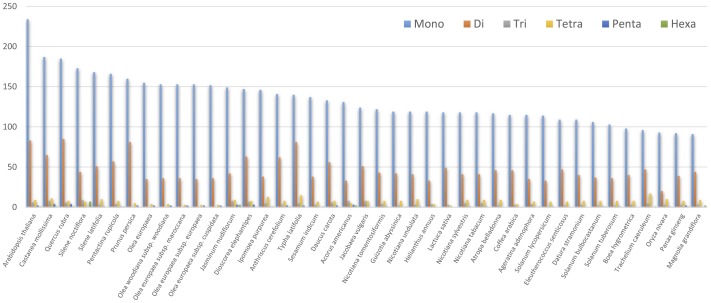
Distribution of SSRs present in the 41 asteridae chloroplast genomes.

The simple sequence repeats (SSR), also called microsatellites, are a group of tandem repeated sequences which consist of 1–6 nucleotide repeat units [38]. A total of 160 SSR loci were detected in *D. stramonium* cp genome including 109 mononucleotide, 40 dinucleotide, 3 trinucleotide and 8 tetranucleotide repeat units. However, only 53 loci were identified in 19 CDS. Among them, 5 genes were found to harbor at least two SSRs, including *atpA*, *ycf3*, *accD*, *rbcL* and *clpP*. We also detected perfect SSRs longer than 8 bp in *D. stramonium* together with 41 other cp genomes to determine whether there was any homology between the isolated SSR fragments and previously reported sequences (Figure 2). *Arabidopsis thaliana* had the maximum amount of SSRs (335) while the smallest number (127) occurred in *Oryza nivara*. Mononucleotide and dinucleotide repeat units are the prevalent types in all species, ranging from 91 (*Magnolia grandiflora*) to 234 (*Arabidopsis thaliana*) and 20 (*Oryza nivara*) to 85 (*Quercus rubra*) in quantity, respectively. The number of trinucleotides is slightly lower than that of tetranucleotides, and only rarely are pentanucleotides or hexanucleotides observed in these 41 cp genomes. Most of SSRs detected in these cp genomes were A (28.2%) and T (35.2%) mononucleotide SSRs while C or G repeats were rarely found. We also detected the distribution of SSRs in the CDS of studied cp genomes (Table S5.). The CDS accounts for approximately 51% of the total length, whereas the SSR proportion ranges from 19% to 41%. Average total number of SSRs identified in CDS is 56 accounting for 30% of all SSRs in these whole cp genomes. In addition, the majority of SSRs are located in LSC region (63.2–66.9%) in 10 Solanales cp genomes.

### Repeat Analysis

For the repeat structure analysis, there are 33 large repeats of 30 bp or larger in *Datura stramonium* cp genome (Table 3). Eleven forward, nine palindromic and thirteen tandem repeats were identified. There are three repeat motifs detected in the CDS of *ycf2* gene and the *IGS* (*rps12.trnV-GAC*)). In 11 repeats there were two repeat motifs while in other 20 repeats only one motif was found (Table 3). Most repeats are located in the intergenic or intronic regions, while some of them are in protein-coding regions. Most of the repeats exhibit lengths between 30 and 60 bp, while the two longest repeats respectively occurred in rrn4.5-rrn5 (66 bp) and IGS (*rps12.trnV-GAC*). Eight forward, six palindromic and eight tandem repeats were distributed in the LSC region.

**Table 3:** Repeated sequences in the *Datura stramonium* chloroplast genome.

Repeat number	Size (bp)	Type	Location	Repeat Unit	Region
1	50	F	*psaB* (CDS), *psaA* (CDS)	GAGAAAAATAAATGCAATAGCTAAATGGTGATGGGCAATATCAGTCAGCC	LSC
2	39	F	*ycf3* (intron), IGS (*rps12*, *trnV-GAC*)	CCAGAACCGTACGTGAGATTTTCACCTCATACGGCTCCT	LSC, IRa
3	39	F	*ycf3* (intron), *ndhA* (intron)	ACAGAACTGTACGTGAGATTTTCACCTCATACGGCTCCT	LSC, SSC
4	39	F	IGS (*rps12*, *trnV-GAC*), *ndhA* (intron)	TCAGAACCGTACATGAGATTTTCACCTCATACGGCTCCT	IRa, SSC
5	40	F	*trnF-GAA*	TCAGAGGACTGAAAATCCTCGTGTTACCACTCCAAATCTG	LSC
6	34	F	IGS (*rrn4.5*, *rrn5*)	TCATTGTTCAAATCTTTGACAACACGAAAAAACC	SSC
7	35	F	*ycf2* (CDS)	AATATTGATGATAGTGACGATATTGATGATAGTGA	IRa
8	30	F	*ycf3* (intron), IGS (*rps12*, *trnV-GAC*)	GTGAGATTTTCACCTCATACGGCTCCTCCC	LSC, IRa
9	31	F	*trnS-GCU*, *trnS-UGA*	CAACGGAAAGAGAGGGATTCGAACCCTCGGT	LSC
10	31	F	*trnG-GCC*, *trnG-UCC*	CGATGCGGGTTCGATTCCCGCTACCCGCTCT	LSC
11	31	F	IGS (*psbC*, *trnS-UGA*), *clpP* (intron)	CTTTTTTCTTTTTTGTTTTCAACTCATTTTA	LSC
12	56	P	*petD* (intron)	GTATAAGTGAACTAGATAAAACGGAATCTTGATTCCGTTTTATCTAGTTCACTTAT	LSC
13	48	P	IGS (*psbT*, *psbN*)	CAGTTGAAGTACTGAGCCTCCCGATATCGGGAGGCTCAGTACTTCAAC	LSC
14	39	P	*ycf3* (intron), IGS (*trnV-GAC*, *rps12*)	CCAGAACCGTACGTGAGATTTTCACCTCATACGGCTCCT	LSC, IRb
15	39	P	*ndhA* (intron), IGS (*trnV-GAC*, *rps12*)	ACAGAACTGTACGTGAGATTTTCACCTCATACGGCTCCT	SSC, IRb
16	30	P	*trnS-GCU*, *trnS-GGA*	AACGGAAAGAGAGGGATTCGAACCCTCGGT	LSC
17	34	P	IGS (*rrn4.5*, *rrn5*), IGS (*rrn5*, *rrn4.5*)	TCATTGTTCAAATCTTTGACAACACGAAAAAACC	IRa, IRb
18	35	P	*ycf2* (CDS)	AATATTGATGATAGTGACGATATTGATGATAGTGA	IRa, IRb
19	32	P	IGS (*trnE-UUC*, *trnT-GGU*)	CTTTTTTTATTTAGAAATTTGTAAATAAAAAA	LSC
20	30	P	*trnS-UGA*, *trnS-GGA*	AAAGGAGAGAGAGGGATTCGAACCCTCGAT	LSC
21	44	T	IGS (*trnK-UUU*, *rps16*)	CTACTTAATTTAAAATTTAAAA (*2)	LSC
22	40	T	IGS (*atpH*, *atpI*)	TTATTCATTTTTATTATTTAT (*2)	LSC
23	33	T	IGS (*rps2*, *rpoC2*)	CATTATTCTTTTCTATT (*2)	LSC
24	45	T	IGS (*trnT-GGU*, *psbD*)	ATTAATTTCATCTATATTATATA (*2)	LSC
25	35	T	IGS (*trnT-UGU*, *trnL-UAA*)	TTCTATATTGGATTCTA (*2)	LSC
26	50	T	IGS (*trnP-GGG*, *psaJ*)	ATTATATAGAAAATACTTATATACA (*2)	LSC
27	41	T	*rps18* (CDS)	TAAATCCAAGCGACCTTTTCT (*2)	LSC
28	47	T	*clpP* (intron)	GATAAAGCAAAGAGAAAAGAA (*2)	LSC
29	58	T	*ycf2* (CDS)	ATATTGATGATAGTGACG (*3)	IRa
30	62	T	IGS (*rps12*, *trnV-GAC*)	TATTATATTAGTATTTTCTATT (*3)	IRa
31	66	T	IGS (*rrn4.5*, *rrn5*)	CATTGTTCAAATCTTTGACAACACGAAAAAAC (*2)	IRa
32	39	T	*ndhF* (CDS)	AATAAAAACCTAAAATTCCT (*2)	SSC
33	55	T	*ycf1* (CDS)	TTCCTTTCTTTGATTCTCCTCTTTTTT (*2)	SSC

*copy number.

‘F’ is forward, ‘P’ is palindromic, and ‘T’ is Tandem; IGS: Intergenic spacer; CDS: protein-coding regions.

### Comparison with Other cp Genomes in the Solanales Order

**Figure 3:**
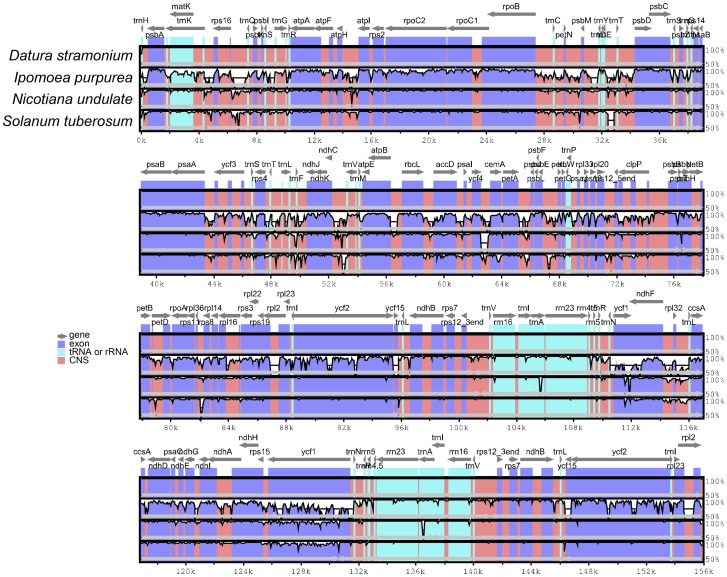
Comparison of four chloroplast genomes using mVISTA program. Grey arrows and thick black lines above the alignment indicate genes with their orientation and the position of the IRs, respectively. A cut-off of 70% identity was used for the plots, and the Y-scale represents the percent identity between 50–100%. Genome regions are color-coded as protein-coding (exon), rRNA, tRNA and conserved noncoding sequences (CNS).

There are currently ten complete cp genome sequences in the Solanales order available in genbank. The gene order and organization of *Datura* are almost identical to those of *N. tabacum* (NC_001879) and other species. The average size of the Solanales cp genomes is 156,422 bp in length. *Ipomoea purpurea* has the largest genome size that is approximately 6.2 kb larger than that of *D. stramonium*, which is mainly attributed to the difference in the length of the IR regions. The genome size of *S. tuberosum* is smallest and is approximately 575 bp smaller than that of *D. stramonium*. This variation in sequence length is mainly caused by the divergence in the length of the LSC region (Table S6). We compared four cp genomes from four different genera in Solanales and observed approximately identical gene order and organization among them (Figure 3). The overall sequence identity of the four cp genomes was plotted using *D. stramonium* as reference. The average sequence divergence of coding regions in *Ipomoea purpurea* is 1.47%, while 1.06% and 1.09% in *Nicotiana undulate* and *Solanum tuberosum*, respectively (Table S7). This study found that the ten most divergent coding regions were *ycf1, clpP, cemA, accD, rpl32, rpl22, matK, ccsA, ndhF* and *rpl36* based on the p-distance measurements. These genes are mainly located in single copy regions. In addition, sequences in non-coding regions exhibit a higher divergence than those in coding regions and the most divergent regions localize in the intergenic spacers among the four cp genomes. In our alignment, these highly divergent regions included *trnH-psbA*, *rps4-trnS*, *ndhD-ccsA* and *ndhI-ndhG*.

**Figure 4:**
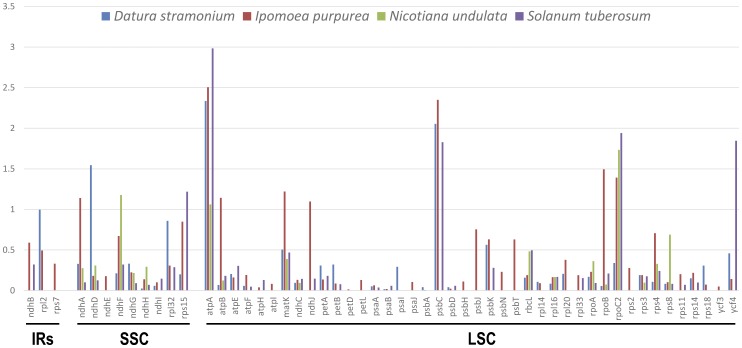
The Ka/Ks ratio of Datura stramonium, Ipomoea purpurea, Nicotiana undulate and Solanum tuberosum for comparison with Nicotiana tabacum.

The non-synonymous (Ka) to synonymous (Ks) rate ratio (denoted by Ka/Ks) among *Datura*, *Ipomoea*, *Nicotiana* and *Solanum* was calculated and is shown in Figure 4. In IRs region, the Ka/Ks ratio of different genes was all lower than that in the SSC and LSC regions. In four species, most of the ratios of genes were below 1.0, except the value of *atpA*, *rpoC2*. The Ka/Ks values of *atpA*, *rpoC2* and *psbC* (except in *Nicotiana undulate*) among four species are all over 1.0, which means the positive selection was exerting an influence on these genes in the evolution of Solanoideae. In contrast, ratios in gene of *Datura stramonium* were variable from 0 to 0.99 (exclude *ndhD*, 1.54), indicating these gene may have already been under purify selection prior to the evolution between *D. stramonium* and *N. tabacum*. *Nicotiana undulate* was relatively closest to the reference species *Nicotiana tabacum* among all four species. The Ka/Ks ratio was 0 in 45 of 62 compared gene, which displayed that the rate of synonymous and non- synonymous was not existed among *N. undulate* and *N. tabacum*. The evolution in species level of Solanoideae is highly conservative.

Variation between the coding sequences of *D. stramonium* and *Ipomoea purpurea*, *Nicotiana undulate* or *Solanum tuberosum* was also analyzed by comparing each individual gene as well as the overall sequences (). The four rRNA genes are the most conserved, while the most divergent coding regions are *accD*, *cemA*, *psbT*, *clpP*, and *ycf1*.

### IR Contraction and Expansion

**Figure 5:**
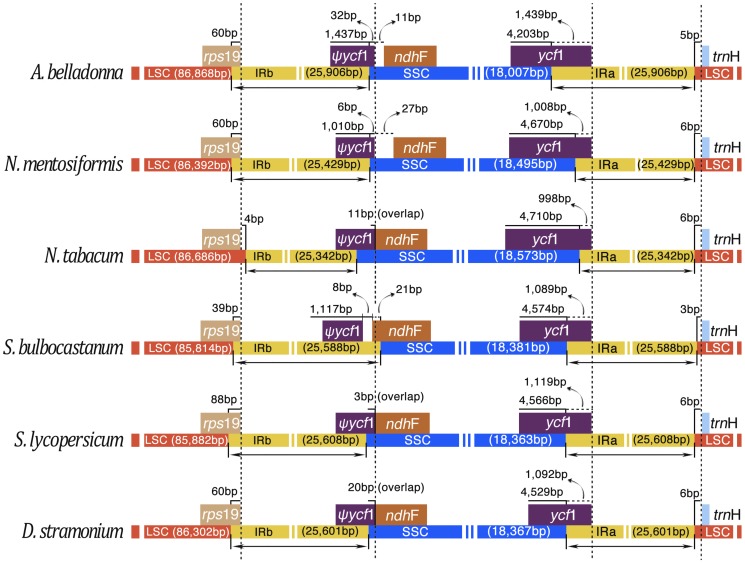
Comparison of the borders of LSC, SSC and IR regions among six chloroplast genomes. The IRb/SSC border extended into the *ycf1* genes to create various lengths of *ycf1* pseudogenes among six chloroplast genomes. The *ycf1* pseudogene and the *ndhF* gene overlapped in the *N. tabacum*, *S. lycopersicum* and *D. stramonium* cp genomes from 3 bp to 20 bp, respectively. Various lengths of *rps19* pseudogenes were created at the IRa/LSC borders except *N. tabacum*. This figure is not to scale.

The size variation of angiosperm cp genomes is primarily due to expansion and contraction of the IR region and the single copy (SC) boundary regions. Detailed comparison at the junction of the IR/SC boundaries among *Atropa belladonna*, *Nicotiana tomentosiformis*, *Nicotiana tabacum*, *Solanum bulbocastanum*, *Solanum lycopersicum*, *Datura stramonium* was presented in Figure 5. Despite the similar length of the IR regions in the six species, from 25,342 bp to 25,906 bp, some IR expansions and contractions were observed. *Rps19* and *ycf1* pseudogenes of various lengths were located at the IRb/LSC and IRb/SSC boundaries, respectively. The border of IRb-LSC junction was located within the *rps19* gene in these cp genomes except in *N. tabacum*, resulting in the formation of the *rps19* pseudogenes. In *D. stramonium*, a short *rps19* pseudogene of 60 bp was created at the IRa-LSC border. The same pseudogene was 60 bp in *A. belladonna* and *N. tomentosiformis*, 39 bp in *S. bulbocastanum* and 88 bp in *S. lycopersicum*, respectively. The IRb-SSC border extended into the *ycf1* genes to create long *ycf1* pseudogenes in all of the cp genomes except in *Solanum bulbocastanum* where the IRb-SSC border expanded to duplicate a part of *ndhF* gene. The length of *ycf1* pseudogene was 1,469 bp in *A. belladonna*, 1,016 bp in *N. tomentosiformis*, 1,052 bp in *N. tabacum*, 1,117 bp in *S. bulbocastanum*, 1,139 bp in *Solanum lycopersicum* and 1,118 bp in *D. stramonium*. In *N. tabacum*, *S. lycopersicum* and *D. stramonium* cp genomes, the *ycf1* pseudogene and the *ndhF* gene overlapped by 11 bp, 3 bp and 20 bp, respectively. The IRa-SSC border was located within the CDS of *ycf1* gene and the length of the part of this gene in IR region was significantly different among the six cp genomes. The *trnH* gene was all located in the LSC regions and has 3–6 bp apart from the IRa-LSC border.

### Phylogenetic Analysis

**Figure 6:**
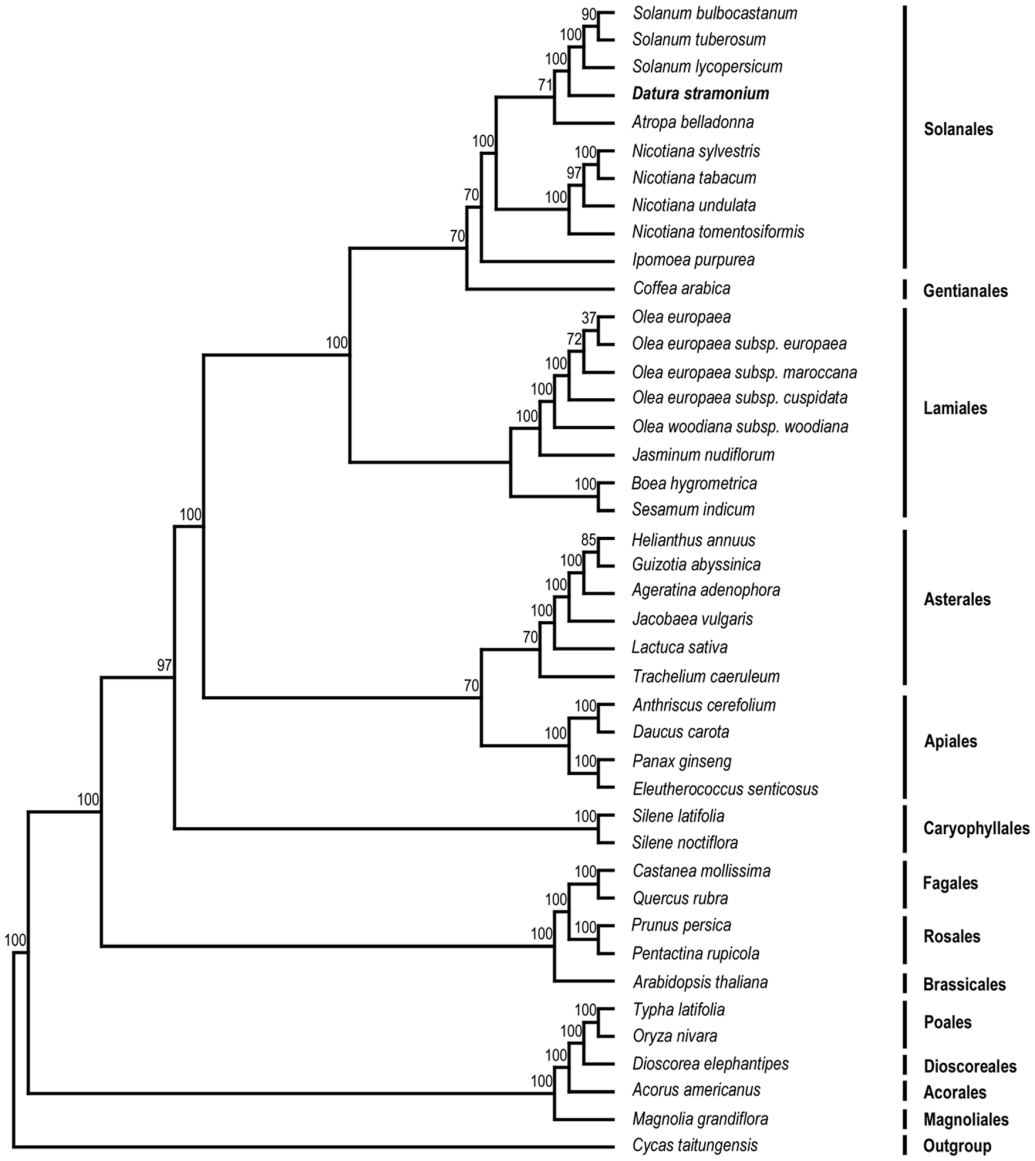
The ML phylogenetic tree of the asteridae clade based on 68 protein-coding genes. The ML tree was obtained with the - lnL of 356757.56 using the GTR+I+G nucleotide substitution model. Number above each node are bootstrap support values. *Cycas taitungensis* was set as outgroup.

Phylogenetic analysis was performed on a 42-taxon 68-gene data matrix using MP and ML methods. The sequence alignment data comprised 41,127 characters after the gaps were excluded to avoid alignment ambiguities due to length variation. The MP analysis resulted in a single most-parsimonious tree (Figure S1) with a consistency index (CI) of 0.53 (excluding uninformative characters) and a retention index (RI) of 0.68. Bootstrap analysis showed that 36 of the 40 nodes were supported by values ≥70%, and all of the nodes had a bootstrap value >50%. The ML analyses, using a single model for all of the genes (GTR +G+I), produced a single tree (Figure 6) with -lnL (unconstrained)  = 356757.56. The ML bootstrap values were also high, with values of ≥90% for 31 of the 39 nodes and only one support value <70%. ML and MP trees exhibited the same topology within asteridae lineage and phylogenetic position of *Datura* was found between *Solanum* and *Atropa* in this study.

## Discussion

### Comparative Analysis of the cp Genome Organization

*Datura stramonium*, also known as jimson weed, devil weed or thorn apple, has been used for mystic and religious purposes as a mystical sacrament which brings about powerful visions and opens the user to communication with spirit world [39]. Especially *D. stramonium* had a long history of medicinal use in Asian countries since two thousand years ago. During the Three Kingdom Period (220–265 A.D.),its use as the first anesthetic for surgery was recorded in literatures [40]. Modern studies showed that *D. stramonium* had varieties of pharmacological effects including antiasthmatic [41], antiepileptic [42], antioxidant [43], antimicrobial [44], [45], antifungal [46] and anti-inflammatory [47] activities. Approximately 400 complete cp genome sequences have been sequenced in GenBank. However most of these sequences are focused on economically important plants, such as *Solanum lycopersicum*, *Oryza sativa* and *Nicotiana tabacum*. In contrast, only few cp genome sequences have been reported for medicinal purposes such as *Salvia miltiorrhiza* and *Panax ginseng*, and still no cp genome sequences have been reported for *Datura*. The availability of the complete cp DNA sequences from *D. stramonium* provides us an improved evolutionary understanding of the chloroplast genome itself and it can also serve as a medicinal improvement tool. The *D. stramonium* cp genome has a typical angiosperm organization with a pair of IRs separating the LSC and SSC regions and exhibits identical gene order and content to the sequenced Solanales cp genomes [48], emphasizing the highly conserved nature of these land plant cp genomes [49]. The cp genome of *D. stramonium* has no significant difference compared with other Solanales genomes except *Ipomoea purpurea* (162,046 bp, Table S6). The GC content could be one of the most important factors in the evolution of genomic structures [50]. We found that the GC content was unevenly distributed in the entire cp genome of *D. stramonium* and the divergence of conserved nature between IR and SC regions might be partly due to the different GC content. In addition, the *ycf15* gene was completely annotated in *D. stramonium* cp genome while most recent studies supported the conclusion that *ycf15* is not a functional gene in protein-coding process [51]–[53].

In the universal genetic code, codons mainly differ at the third position. Though many synonymous codons needed to regulate the translation process, but only particular codons are preferred. Results in this study showed that the synonymous codons usage was not at the same frequencies and the patterns of synonymous codon usage also varied significantly among genes, which were consistent with previous investigations [54]. Codon bias of cp genes has been reported to be towards codons ended with A or T due to the compositional bias towards AT rich content [55], [56]. Since all cp genomes have high AT content, AT biased mutational pressure is believed to be the factor responsible for codon usage bias. Previous studies demonstrated that there existed a significant relationship between codon usage bias and gene expression level [57], [58], which suggested stronger natural selection constraints on highly expressed genes to optimize translation efficiency using major codons [59]. Information about the rare and preferred codons can be effectively used for enhancing gene expression by optimizing synonymous codons, which may provide us a further understanding of synthesis and metabolism of secondary metabolites in *D. stramonium*.

The intron plays an important role in the regulation of gene expression. Some recent studies have found that many introns improve exogenous gene expression at specific positions and times, resulting in the expected agronomic characters. Therefore, introns can be a useful tool to improve transformation efficiency [60]. A total of 14 intron-containing genes were detected and 11 of which contain one intron but 3 of which have two introns, which are similar to the cp genome of *Nicotiana tabacum*. These results are helpful for further transformation studies in *D. stramonium*.

Cp SSRs have frequently been used in species identification and genetic analysis at individual or group levels because of their high reproductivity, codominant inheritance, relatively high polymorphism, and relative abundance in genomes. There are altogether 160 cp SSRs discovered in *D. stramonium* cp genome. These markers will allow us to improve our understanding of the population structure and genetic diversity of this species that are essential for molecular breeding and cp genetic engineering. In this study, we also investigated the distribution of SSR in 41 cp genomes of Asteridae. The average number of SSRs in the CDS regions accounted for 30% of all discovered SSRs and the average SSR proportion located in LSC regions was 64.95% in these studied cp genomes. This result indicates that SSRs are less abundant in CDS than in non-coding regions and that they are unevenly distributed within Lamiales cp genomes, which provides more information for choosing effective molecular markers and detecting both intra- and interspecific polymorphisms within this order [61], [62]. In addition, mononucleotide, dinucleotide, and trinucleotide repeats were composed of A or T at a higher level. This may contribute to a bias in base composition, which was consistent with the overall A-T richness (62.3%) of the Asteridae cp genome. The bias may have a close relationship with the easier changes to A-T rather than G-C in the genome [63]. An interesting finding was that the first seven SSR loci with largest number of mononucleotide repeat were distributed in Fagales, Rosales, Caryophyllales and Brassicales, and the four groups were closely related within asteridae and formed into a clade in maximum parsimony tree in this study.

In the analysis of repeat structure, 11 forward, 9 palindromic and 13 tandem repeats were revealed. Among these repeats, 72.7% of all forward repeats were distributed in the LSC region, 66.7% and 61.5% in palindromic and tandem repeats, respectively. In addition, most of all repeats are discovered located in the intergenic spacers or introns (Table 3). Short dispersed repeats are considered to be one of the major factors promoting cp genome rearrangements [64]. It was demonstrated that there existed a correlation between the abundance of short dispersed repeats and the extent of gene rearrangements [65]. Most of these repeats always occur near the rearrangements hotspots and may mediate these regions [66], [67]. In addition, short repeat motifs may facilitate inter-molecular recombination and create diversity of chloroplast genomes in a population [68]. Therefore repeats found in this study provide valuable information for phylogeny of *Datura* and population studies of *D. stramonium*.

Differences in cp genome size are mainly caused by the contraction and expansion of the IR regions [63], [69]. However comparison of the IR boundary among six Solanaceae species showed that the size of the IR regions has no significant relationship with the length of the complete cp genome sequence (Figure 5). Correlation analysis indicated that the length of the IR regions had a positive correlation with that of ycf1 gene located in IR region (R2 = 0.9, P<0.05). All *trnH* genes in the six Solanaceae species were found located in LSC region whereas this gene was completely located in the IR region in monocot cp genomes [70]. We also compared four Solanales cp genomes using mVISTA and observed an approximately identical gene order and organization among them (Figure 3). The comparison demonstrates that the two IR regions are less divergent than the LSC and SSC regions. The five most divergent coding regions are *accD*, *cemA*, *psbT*, *clpP* and *ycf1*. The *ycf1* gene is considered as the most variable locus with unknown function in recent study, and is confirmed that it was more variable than the *matK* gene in the Orchidaceae [71]. The *ψycf1* (pseudogenes) located in the IRb region is conservative while the *ycf1* located in the SSC with highly variable. Dong *et al* used the two regions of *ycf1* (*ycf1*-a and *ycf1*-b) as a new tool to solve the phylogenetic problems at species level and for DNA barcoding of some closely related flowering plant species because of their high variability [72]. In addition, non-coding regions exhibit a higher divergence than coding regions and the most divergent regions localize in the intergenic spacers. These highly divergent regions including *trnH-psbA*, *rps4-trnS*, *ndhD-ccsA* and *ndhI-ndhG* can be used to develop markers or specific barcodes [73] that would maximize the ability to differentiate species within the Solanales. Data analysis concerning sequence divergence (Table S7) and Ka/Ks ratio (Figure 4) also supported that the IR regions are more conserved compared with SSC and LSC regions.

### Phylogenetic Implications

Chloroplast genomes have shown a substantial power in studies of phylogenetics, evolution and molecular systematics. During the last decade, there have been many analyses to address phylogenetic questions at deep nodes based on comparison of multiple protein-coding genes [74]–[76] and complete sequences in chloroplast genomes [77], [78], enhancing our understanding of enigmatic evolutionary relationships among angiosperms. However, further development of Asteridae phylogeny is typically limited due to sporadic taxon sampling. Phylogenetic analysis using maximum likelihood and maximum parsimony were performed based on 68 shared genes (Table S2) in 42 sequenced genomes, including the cp genome of *D. stramonium* sequenced in this study, to examine the position of *D. stramonium* and relationships within the Asteridae. Both trees have provided strong support for the position of *D. stramonium* as a sister to *Solanum lycopersicum*, followed by *Atropa belladonna* (Figure 6 and Figure S1). The difference between the MP and ML trees involves the position of *Silene*, which is likely to be caused by long-branch attraction [79]. The asteridae, the largest and diverse subclass in angiosperm, includes more than 60,000 species and is widely distributed throughout the world. More taxon samplings should be required to clarify accurate relationships among asteridae.

### Implication for Chloroplast Genetic Engineering

Chloroplasts are distributed throughout the differentiated cells of plant organs and tissues. Over evolutionary time, cp genomes have given up most of their genes and cellular functions to become the energy transduction and metabolic center of plant cell [80]. The high copy number of chloroplast genomes makes it possible to provide an engineering of multiple foreign genes for the production of a metabolic pathway with a high transformation rate in contrast with nuclear transformation. Great progress in chloroplast engineering has been achieved since the first chloroplast genetic transformation succeeded two decades ago [81].

Although a number of plant species are transformable, plastid transformation is now routinely carried out only in tobacco [82]. In addition, while gene regulation is generally conserved, expression of a foreign gene may vary between different plant species [83]. The expression of a transgene can be affected by various factors such as the promoter, the 5′ untranslated region (UTR), the downstream box, the N-terminal amino acid sequence, the codon usage, the 3′ UTR and genes located upstream and downstream [83]. The efficiency of transformation in most plants remains too low. This study showed that *Datura stramonium* had an identical plastid genome structure and similar sequence relative to *Nicotiana tabacum*. The two plant species are very closely related in evolutionary relationship. In addition, many transformable species are from Solanaceous including tomato [84], petunia [85], eggplant [86] and potato [87]. *D*. *stramonium* may have great potential to become a model medicinal plant to carry out plant transformation. The availability of the complete cp genome sequence of *D*. *stramonium* is helpful to recognize the optimal regions for transgene integration and to develop site-specific cp transformation vectors.

*Datura stramonium* naturally grows in warm and temperate regions and does have a low tolerate for cold environments. They are not especially susceptible to pests, but will suffer from mealy bugs and aphids. In addition, *Datura* are propagated by seed. Young seedlings are very tolerant of poor soil and even drought but cannot tolerate herbicide. The plastid genome is an attractive location for the engineering of pest-resistance and herbicide-tolerance traits. Expression of insecticidal proteins and herbicide-tolerant enzymes from the chloroplast genome has proven to be a very efficient strategy for successful resistance management and weed control [88]–[92]. Plastid engineering should be particularly useful to develop resistant to abiotic and biotic stresses in molecular breeding of *D. stramonium*.

## Conclusions

This is the first study of complete cp genome of *Datura* species which can extract scopolamine. The gene order and genome organization of *D. stramonium* are similar to those of cp genomes in the Solanales. There are no significant structural rearrangements of Solanales cp genomes during the evolutionary process. Further, the repeated sequences, SSR and protein-coding gene sequence were determined. Phylogenetic relationships among 42 angiosperms provide a strong support for the position of *D. stramonium*. In addition, the data presented in this paper will facilitate the further biological study in the field of phylogenomics and plant biotechnology of this important poisonous and medicinal plant.

## Supporting Information

Figure S1 The MP phylogenetic tree of the asteridae clade based on 68 protein-coding genes. The MP tree has a length of 59,852, with a consistency index of 0.53 and a retention index of 0.68. Number above each node are bootstrap support values. *Cycas taitungensis* was set as outgroup.
(TIF)

Table S1 The list of accession numbers of the chloroplast genome sequences used in this study.
(DOC)

Table S2 Average pairwise sequence distance of protein-coding genes among 42 chloroplast genomes.
(DOC)

Table S3 Genes present in the Datura stramonium chroloplast genome.
(DOC)

Table S4 The genes with introns in the Datura stramonium chloroplast genome and the length of the exons and introns.
(DOC)

Table S5 Distribution of SSRs present in the CDS among 41 asteridae chloroplast genomes.
(DOC)

Table S6 Size comparison of 10 cp genomes in the order of Solanales.
(DOC)

Table S7 Comparison of homologues between the *Datura stramonium* and *Ipomoea purpurea* (*Ip*), *Nicotiana undulate* (*Nu*) or *Solanum tuberosum* (*St*) chloroplast genomes using the percent identity of protein-coding sequences.
(DOC)
