# Correction: Complete Chloroplast Genome Sequence of Poisonous and Medicinal Plant *Datura stramonium*: Organizations and Implications for Genetic Engineering

**DOI:** 10.1371/journal.pone.0118236

**Published:** 2015-02-06

**Authors:** The PLOS ONE Staff

The authors’ first and last names were inadvertently switched in the byline. The first name should be the last name and the last name should be the first for all authors.

The correct byline is:

Yang Yang, Yuanye Dang, Qing Li, Jinjian Lu, Xiwen Li, Yitao Wang.

The correct citation is:

Yang Y, Dang Y, Li Q, Lu J, Li X, et al. (2014) Complete Chloroplast Genome Sequence of Poisonous and Medicinal Plant *Datura stramonium*: Organizations and Implications for Genetic Engineering. PLoS ONE 9(11): e110656. doi:10.1371/journal.pone.0110656
