# Supplementary figures and images for: Complete Chloroplast Genome Sequence of Poisonous and Medicinal Plant Datura stramonium: Organizations and Implications for Genetic Engineering
Source: PLoS One. 2014 Nov 3;9(11):e110656. doi: 10.1371/journal.pone.0110656 (PMC4217734; doi:10.1371/journal.pone.0110656)

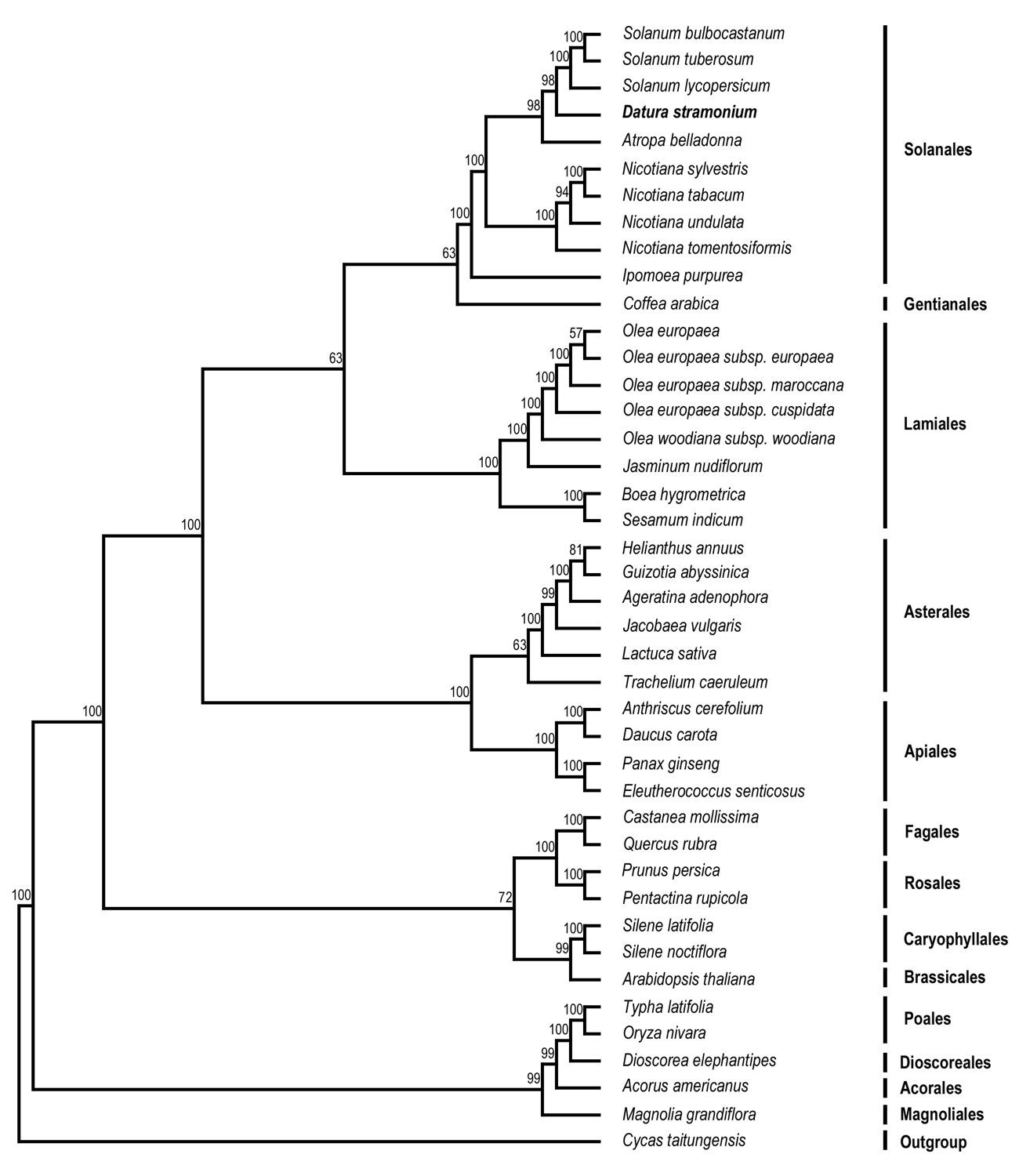

Supplement: Figure S1 — The MP phylogenetic tree of the asteridae clade based on 68 protein-coding genes. The MP tree has a length of 59,852, with a consistency index of 0.53 and a retention index of 0.68. Number above each node are bootstrap support values. Cycas taitungensis was set as outgroup. (TIF) [file pone.0110656.s001.tif]
