# Supplementary material for: Complete Chloroplast Genome Sequence of Poisonous and Medicinal Plant Datura stramonium: Organizations and Implications for Genetic Engineering
Source: PLoS One. 2014 Nov 3;9(11):e110656. doi: 10.1371/journal.pone.0110656 (PMC4217734; doi:10.1371/journal.pone.0110656)
Supplement: Table S1 — The list of accession numbers of the chloroplast genome sequences used in this study. (DOC) [file pone.0110656.s002.doc]

**Table S1.** The list of accession numbers of the chloroplast genome sequences used in this study.

| No. | Taxon | Family | Order | GenBank Accession number |
| --- | --- | --- | --- | --- |
| 1 | *Datura stramonium* | Solanaceae | Solanales | NC_018117 |
| 2 | *Anthriscus cerefolium* | Apiaceae | Apiales | NC_015113 |
| 3 | *Daucus carota* | Apiaceae | Apiales | NC_008325 |
| 4 | *Eleutherococcus senticosus* | Araliaceae | Apiales | NC_016430 |
| 5 | *Panax ginseng* | Araliaceae | Apiales | NC_006290 |
| 6 | *Ageratina adenophora* | Asteraceae | Asterales | NC_015621 |
| 7 | *Guizotia abyssinica* | Asteraceae | Asterales | NC_010601 |
| 8 | *Helianthus annuus* | Asteraceae | Asterales | NC_007977 |
| 9 | *Jacobaea vulgaris* | Asteraceae | Asterales | NC_015543 |
| 10 | *Lactuca sativa* | Asteraceae | Asterales | NC_007578 |
| 11 | *Trachelium caeruleum* | Asteraceae | Asterales | NC_010442 |
| 12 | *Coffea arabica* | Rubiaceae | Gentianales | NC_008535 |
| 13 | *Boea hygrometrica* | Gesneriaceae | Lamiales | NC_016468 |
| 14 | *Jasminum nudiflorum* | Oleaceae | Lamiales | NC_008407 |
| 15 | *Olea europaea* | Oleaceae | Lamiales | NC_013707 |
| 16 | *Olea europaea subsp. cuspidata* | Oleaceae | Lamiales | NC_015604 |
| 17 | *Olea europaea subsp. europaea* | Oleaceae | Lamiales | NC_015401 |
| 18 | *Olea europaea subsp. maroccana* | Oleaceae | Lamiales | NC_015623 |
| 19 | *Olea woodiana subsp. woodiana* | Oleaceae | Lamiales | NC_015608 |
| 20 | *Sesamum indicum* | Pedaliaceae | Lamiales | NC_016433 |
| 21 | *Atropa belladonna* | Solanaceae | Solanales | NC_004561 |
| 22 | *Ipomoea purpurea* | Convolvulaceae | Solanales | NC_009808 |
| 23 | *Nicotiana sylvestris* | Solanaceae | Solanales | NC_007500 |
| 24 | *Nicotiana tabacum* | Solanaceae | Solanales | NC_001879 |
| 25 | *Nicotiana tomentosiformis* | Solanaceae | Solanales | NC_007602 |
| 26 | *Nicotiana undulata* | Solanaceae | Solanales | NC_016068 |
| 27 | *Solanum bulbocastanum* | Solanaceae | Solanales | NC_007943 |
| 28 | *Solanum lycopersicum* | Solanaceae | Solanales | NC_007898 |
| 29 | *Solanum tuberosum* | Solanaceae | Solanales | NC_008096 |
| 30 | *Arabidopsis thaliana* | Brassicaceae | Brassicales | NC_000932 |
| 31 | *Castanea mollissima* | Fagaceae | Fagales | NC_014674 |
| 32 | *Magnolia grandiflora* | Magnoliaceae | Magnoliales | NC_020318 |
| 33 | *Pentactina rupicola* | Rosaceae | Rosales | NC_016921 |
| 34 | *Prunus persica* | Rosaceae | Rosales | NC_014697 |
| 35 | *Quercus rubra* | Fagaceae | Fagales | NC_020152 |
| 36 | *Silene latifolia* | Caryophyllaceae | Caryophyllales | NC_016730 |
| 37 | *Silene noctiflora* | Caryophyllaceae | Caryophyllales | NC_016728 |
| 38 | *Acorus americanus* | Acoraceae | Acorales | NC_010093 |
| 39 | *Dioscorea elephantipes* | Dioscoreaceae | Dioscoreales | NC_009601 |
| 40 | *Oryza nivara* | Poaceae | Poales | NC_005973 |
| 41 | *Typha latifolia* | Typhaceae | Poales | NC_013823 |
| 42 | *Cycas taitungensis* | Cycadaceae | Cycadeles | NC_009618 |
