# Supplementary material for: Complete Chloroplast Genome Sequence of Poisonous and Medicinal Plant Datura stramonium: Organizations and Implications for Genetic Engineering
Source: PLoS One. 2014 Nov 3;9(11):e110656. doi: 10.1371/journal.pone.0110656 (PMC4217734; doi:10.1371/journal.pone.0110656)
Supplement: Table S3 — Genes present in the Datura stramonium chroloplast genome. (DOC) [file pone.0110656.s004.doc]

**Table S3.** Genes present in the *Datura stramonium* chroloplast genome.

|  | Group of genes | Gene names |
| --- | --- | --- |
| 1 | Photosystem I | *psaA*, *psaB*, *psaC*, *psaI*, *psaJ* |
| 2 | Photosystem II | *psbA*, *psbB*, *psbC*, *psbD*, *psbE*, *psbF*, *psbH*, *psbI*, *psbJ*, *psbK*, *psbL*, *psbM*, *psbN*, *psbT*, *psbZ* |
| 3 | Cytochrome b/f complex | *petA*, *petB**, *petD**, *petG*, *petL*, *petN* |
| 4 | ATP synthase | *atpA*, *atpB*, *atpE*, *atpF**, *atpH*, *atpI* |
| 5 | NADH dehydrogenase | *ndhA**, *ndhB**(×2), *ndhC*, *ndhD*, *ndhE*, *ndhF*, *ndhG*, *ndhH*, *ndhI*, *ndhJ*, *ndhK* |
| 6 | RubisCO large subunit | *rbcL* |
| 7 | RNA polymerase | *rpoA*, *rpoB*, *rpoC1**, *rpoC2* |
| 8 | Ribosomal proteins (SSU) | *rps2*, *rps3*, *rps4*, *rps7*(×2), *rps8*, *rps11*, *rps12***(×2), *rps14*, *rps15*, *rps16**, *rps18*, *rps19* |
| 9 | Ribosomal proteins (LSU) | *rpl2**(×2), *rpl14*, *rpl16**, *rpl20*, *rpl22*, *rpl23*(×2), *rpl32*, *rpl33*, *rpl36* |
| 10 | Other genes | *clpP***, *matK*, *accD*, *ccsA*, *infA*, *cemA* |
| 11 | Proteins of unknown function | *ycf1*(×2), *ycf2*(×2), *ycf3***, *ycf4*, *ycf15*(×2) |
| 12 | Transfer RNAs | 29 *tRNA*s (2 contain an intron, 7 in the IRs) |
| 13 | Ribosomal RNAs | *rrn4.5*(×2), *rrn5*(×2), *rrn16*(×2), *rrn23*(×2) |

One or two asterisks after genes indicate that gene contains one or two introns, respectively.
