# Supplementary material for: Complete Chloroplast Genome Sequence of Poisonous and Medicinal Plant Datura stramonium: Organizations and Implications for Genetic Engineering
Source: PLoS One. 2014 Nov 3;9(11):e110656. doi: 10.1371/journal.pone.0110656 (PMC4217734; doi:10.1371/journal.pone.0110656)
Supplement: Table S4 — The genes with introns in the Datura stramonium chloroplast genome and the length of the exons and introns. (DOC) [file pone.0110656.s005.doc]

**Table S4.** The genes with introns in the *Datura stramonium* chloroplast genome and the length of the exons and introns.

| Gene | Location | Exon I (bp) | Intron I (bp) | Exon II (bp) | Intron II (bp) | Exon III (bp) |
| --- | --- | --- | --- | --- | --- | --- |
| *trnL-UAA* | LSC | 34 | 502 | 49 |  |  |
| *trnV-UAC* | LSC | 36 | 570 | 37 |  |  |
| *rps12** | LSC | 114 | - | 231 | 537 | 30 |
| *rps16* | LSC | 226 | 867 | 40 |  |  |
| *rpl2* | IR | 428 | 670 | 392 |  |  |
| *rpl16* | LSC | 395 | 1026 | 8 |  |  |
| *atpF* | LSC | 409 | 701 | 144 |  |  |
| *petB* | LSC | 5 | 747 | 641 |  |  |
| *petD* | LSC | 9 | 749 | 473 |  |  |
| *ndhA* | SSC | 539 | 1155 | 551 |  |  |
| *ndhB* | IR | 755 | 680 | 776 |  |  |
| *ycf3* | LSC | 158 | 754 | 229 | 741 | 123 |
| *clpP* | LSC | 233 | 625 | 291 | 793 | 70 |
| *rpoC1* | LSC | 1613 | 738 | 452 |  |  |

The *rps12* is a trans-spliced gene with the 5’ end located in the LSC region and the duplicated 3’ end in the IR regions.
