# Supplementary material for: Complete Chloroplast Genome Sequence of Poisonous and Medicinal Plant Datura stramonium: Organizations and Implications for Genetic Engineering
Source: PLoS One. 2014 Nov 3;9(11):e110656. doi: 10.1371/journal.pone.0110656 (PMC4217734; doi:10.1371/journal.pone.0110656)
Supplement: Table S5 — Distribution of SSRs present in the CDS among 41 asteridae chloroplast genomes. (DOC) [file pone.0110656.s006.doc]

**Table S5.** Distribution of SSRs present in the CDS among 41 asteridae chloroplast genomes.

| **Taxon** | **Genome Size (bp)** | **AT (%)** | **CDS** | | |
| --- | --- | --- | --- | --- | --- |
|  |  |  | **% ^a^** | **No. ^b^** | **% ^c^** |
| *Acorus americanus* | 153,819 | 61 | 50 | 49 | 27 |
| *Ageratina adenophora* | 150,698 | 63 | 49 | 50 | 31 |
| *Anthriscus cerefolium* | 154,719 | 63 | 50 | 68 | 31 |
| *Arabidopsis thaliana* | 154,478 | 64 | 51 | 84 | 25 |
| *Atropa belladonna* | 156,687 | 62 | 51 | 50 | 29 |
| *Boea hygrometrica* | 153,493 | 62 | 52 | 46 | 31 |
| *Castanea mollissima* | 160,799 | 63 | 49 | 56 | 20 |
| *Coffea arabica* | 155,189 | 63 | 51 | 57 | 34 |
| *Datura stramonium* | 155,871 | 62 | 52 | 53 | 33 |
| *Daucus carota* | 155,911 | 62 | 50 | 69 | 34 |
| *Dioscorea elephantipes* | 152,609 | 63 | 52 | 62 | 27 |
| *Eleutherococcus senticosus* | 156,768 | 62 | 50 | 56 | 34 |
| *Guizotia abyssinica* | 151,762 | 62 | 52 | 71 | 41 |
| *Helianthus annuus* | 151,104 | 62 | 51 | 63 | 39 |
| *Ipomoea purpurea* | 162,046 | 63 | 53 | 67 | 33 |
| *Jacobaea vulgaris* | 150,689 | 63 | 51 | 55 | 29 |
| *Jasminum nudiflorum* | 165,121 | 62 | 50 | 81 | 38 |
| *Lactuca sativa* | 152,765 | 62 | 48 | 43 | 25 |
| *Magnolia grandiflora* | 159,623 | 61 | 49 | 37 | 25 |
| *Nicotiana sylvestris* | 155,941 | 62 | 54 | 62 | 36 |
| *Nicotiana tabacum* | 155,943 | 62 | 54 | 62 | 36 |
| *Nicotiana tomentosiformis* | 155,745 | 62 | 54 | 58 | 33 |
| *Nicotiana undulata* | 155,863 | 62 | 56 | 62 | 36 |
| *Olea europaea* | 155,888 | 62 | 51 | 50 | 26 |
| *Olea europaea subsp. cuspidata* | 155,862 | 62 | 51 | 46 | 24 |
| *Olea europaea subsp. europaea* | 155,875 | 62 | 51 | 46 | 24 |
| *Olea europaea subsp. maroccana* | 155,896 | 62 | 51 | 46 | 24 |
| *Olea woodiana subsp. woodiana* | 155,942 | 62 | 51 | 45 | 23 |
| *Oryza nivara* | 134,494 | 61 | 51 | 42 | 33 |
| *Panax ginseng* | 156,318 | 62 | 50 | 54 | 37 |
| *Pentactina rupicola* | 156,612 | 63 | 50 | 61 | 26 |
| *Prunus persica* | 157,790 | 63 | 50 | 62 | 25 |
| *Quercus rubra* | 161,304 | 63 | 50 | 54 | 19 |
| *Sesamum indicum* | 153,324 | 62 | 51 | 54 | 29 |
| *Silene latifolia* | 151,736 | 64 | 51 | 60 | 26 |
| *Silene noctiflora* | 151,639 | 63 | 54 | 89 | 37 |
| *Solanum bulbocastanum* | 155,371 | 62 | 51 | 48 | 31 |
| *Solanum lycopersicum* | 155,461 | 62 | 51 | 46 | 29 |
| *Solanum tuberosum* | 155,296 | 62 | 51 | 46 | 31 |
| *Trachelium caeruleum* | 162,321 | 62 | 43 | 44 | 27 |
| *Typha latifolia* | 161,572 | 63 | 49 | 59 | 24 |
| Average |  |  | 51 | 56 | 30 |

CDS: protein-coding regions.

^a^Percentage were calculated according to the total length of the CDS divided by the genome size.

^b^Total number of SSRs identified in the CDS.

^c^Percentage were calculated according to the total number of SSRs in the CDS divided by the total number of SSRs in the genome.
