# Supplementary material for: Complete Chloroplast Genome Sequence of Poisonous and Medicinal Plant Datura stramonium: Organizations and Implications for Genetic Engineering
Source: PLoS One. 2014 Nov 3;9(11):e110656. doi: 10.1371/journal.pone.0110656 (PMC4217734; doi:10.1371/journal.pone.0110656)
Supplement: Table S6 — Size comparison of 10 cp genomes in the order of Solanales. (DOC) [file pone.0110656.s007.doc]

**Table S6. Size comparison of 10 cp genomes in the order of Solanales**

| Species | Length (bp) | | | |
| --- | --- | --- | --- | --- |
|  | Total genome | LSC | SSC | IR |
| *Datura stramonium* | 155,871 | 86,302 | 18,367 | 25,601 |
| *Atropa belladonna* | 156,687 | 86,870 | 18,009 | 25,904 |
| *Ipomoea purpurea* | 162,046 | 88,173 | 12,111 | 30,881 |
| *Nicotiana sylvestris* | 155,941 | 86,685 | 18,572 | 25,342 |
| *Nicotiana tabacum* | 155,943 | 86,687 | 18,572 | 25,342 |
| *Nicotiana tomentosiformis* | 155,745 | 86,393 | 18,496 | 25,428 |
| *Nicotiana undulate* | 155,863 | 86,634 | 18,569 | 25,330 |
| *Solanum bulbocastanum* | 155,371 | 85,815 | 18,382 | 25,587 |
| *Solanum lycopersicum* | 155,461 | 85,883 | 18,364 | 25,607 |
| *Solanum tuberosum* | 155,296 | 85,738 | 18,374 | 25,592 |
