# Supplementary material for: Complete Chloroplast Genome Sequence of Poisonous and Medicinal Plant Datura stramonium: Organizations and Implications for Genetic Engineering
Source: PLoS One. 2014 Nov 3;9(11):e110656. doi: 10.1371/journal.pone.0110656 (PMC4217734; doi:10.1371/journal.pone.0110656)
Supplement: Table S7 — Comparison of homologues between the Datura stramonium and Ipomoea purpurea (Ip), Nicotiana undulate (Nu) or Solanum tuberosum (St) chloroplast genomes using the percent identity of protein-coding sequences. (DOC) [file pone.0110656.s008.doc]

**Table S7. Comparison of homologues between the *Datura stramonium* and *Ipomoea purpurea* (*Ip*), *Nicotiana undulate* (*Nu*) or *Solanum tuberosum* (*St*) chloroplast genomes using the percent identity of protein-coding sequences.**

| Gene | Start | End | Length (bp) | % identity | | | |
| --- | --- | --- | --- | --- | --- | --- | --- |
|  |  |  |  | *Ip* | | *Nu* | *St* |
| *accD* | 59310 | 60842 | 1533 | 94.83 | 95.78 | | 97.14 |
| *atpA* | 10430 | 11953 | 1524 | 97.97 | 98.10 | | 98.10 |
| *atpB* | 54788 | 56284 | 1497 | 98.93 | 99.06 | | 98.86 |
| *atpE* | 54390 | 54791 | 402 | 98.76 | 99.25 | | 98.76 |
| *atpF* | 12008 | 12417 | 555 | 98.78 | 99.31 | | 98.54 |
|  | 13118 | 13262 | - | - | - | | - |
| *atpH* | 13664 | 13909 | 246 | 99.59 | 99.19 | | 99.19 |
| *atpI* | 15104 | 15847 | 744 | 98.92 | 99.60 | | 99.46 |
| *ccsA* | 116199 | 117140 | 942 | 97.24 | 97.66 | | 97.98 |
| *cemA* | 63245 | 63934 | 690 | 89.58 | 97.83 | | 98.26 |
| *clpP* | 72167 | 72400 | 597 | 92.81 | 96.58 | | 95.55 |
|  | 73025 | 73316 | - | - | - | | - |
|  | 74109 | 74179 | - | - | - | | - |
| *matK* | 2074 | 3603 | 1530 | 97.12 | 97.84 | | 97.84 |
| *ndhA* | 121541 | 122080 | 1092 | 98.55 | 98.91 | | 98.91 |
|  | 123235 | 123786 | - | - | - | | - |
| *ndhB* | 143278 | 144054 | 1533 | 99.87 | 99.74 | | 99.87 |
|  | 144734 | 145489 | - | - | - | | - |
| *ndhC* | 51808 | 52170 | 363 | 99.45 | 98.62 | | 99.17 |
| *ndhD* | 117377 | 118852 | 1476 | 98.37 | 97.90 | | 98.24 |
| *ndhE* | 119495 | 119800 | 306 | 97.38 | 98.37 | | 98.69 |
| *ndhF* | 111905 | 114127 | 2223 | 97.08 | 97.98 | | 97.89 |
| *ndhG* | 120024 | 120554 | 531 | 98.31 | 98.68 | | 98.87 |
| *ndhH* | 123788 | 124969 | 1182 | 98.14 | 98.31 | | 98.98 |
| *ndhI* | 120953 | 121456 | 504 | 99.21 | 98.21 | | 98.81 |
| *ndhJ* | 50492 | 50968 | 477 | 98.11 | 99.58 | | 99.16 |
| *ndhK* | 51074 | 51817 | 744 | 98.66 | 98.79 | | 98.92 |
| *petA* | 64154 | 65116 | 963 | 98.65 | 98.96 | | 98.96 |
| *petB* | 77125 | 77130 | 648 | 99.22 | 99.38 | | 99.07 |
|  | 77877 | 78518 | - | - | - | | - |
| *petD* | 78709 | 78717 | 483 | 98.73 | 98.52 | | 99.37 |
|  | 79466 | 79939 | - | - | - | | - |
| *petG* | 68262 | 68375 | 114 | 100.00 | 100.00 | | 100.00 |
| *petL* | 67984 | 68079 | 96 | 98.96 | 100.00 | | 100.00 |
| *petN* | 29396 | 29485 | 90 | 100.00 | 100.00 | | 98.89 |
| *psaA* | 41060 | 43312 | 2253 | 99.25 | 99.25 | | 99.38 |
| *psaB* | 38830 | 41034 | 2205 | 99.23 | 99.32 | | 99.18 |
| *psaC* | 118998 | 119243 | 246 | 98.37 | 97.97 | | 98.78 |
| *psaI* | 61437 | 61547 | 111 | 100.00 | 100.00 | | 99.05 |
| *psaJ* | 69258 | 69392 | 135 | 100.00 | 98.52 | | 100.00 |
| *psbA* | 498 | 1559 | 1062 | 99.15 | 99.53 | | 99.25 |
| *psbB* | 74625 | 76151 | 1527 | 99.21 | 99.41 | | 99.02 |
| *psbC* | 35320 | 36741 | 1422 | 98.87 | 99.16 | | 99.30 |
| *psbD* | 34311 | 35372 | 1062 | 99.34 | 99.25 | | 99.25 |
| *psbE* | 66578 | 66829 | 252 | 98.81 | 99.21 | | 99.60 |
| *psbF* | 66449 | 66568 | 120 | 99.17 | 99.17 | | 99.17 |
| *psbH* | 76774 | 76995 | 222 | 97.75 | 99.10 | | 98.65 |
| *psbI* | 8366 | 8476 | 111 | 100.00 | 100.00 | | 100.00 |
| *psbJ* | 66063 | 66185 | 123 | 100.00 | 100.00 | | 99.19 |
| *psbK* | 7816 | 8001 | 186 | 99.36 | 99.46 | | 98.39 |
| *psbL* | 66310 | 66426 | 117 | 100.00 | 99.13 | | 100.00 |
| *psbM* | 30605 | 30709 | 105 | 99.05 | 100.00 | | 100.00 |
| *psbN* | 76531 | 76662 | 132 | 100.00 | 100.00 | | 100.00 |
| *psbT* | 76353 | 76457 | 105 | 97.14 | 99.05 | | 100.00 |
| *psbZ* | 37429 | 37617 | 189 | 98.94 | 99.47 | | 99.47 |
| *rbcL* | 57105 | 58538 | 1434 | 99.02 | 98.88 | | 99.16 |
| *rpl14* | 82854 | 83222 | 369 | 98.92 | 98.92 | | 98.92 |
| *rpl16* | 83347 | 83742 | 405 | 97.98 | 98.99 | | 98.74 |
|  | 84768 | 84776 | - | - | - | | - |
| *rpl2* | 154243 | 154641 | 807 | 100.00 | 99.26 | | 100.00 |
|  | 155326 | 155733 | - | - | - | | - |
| *rpl20* | 70719 | 71105 | 387 | 98.97 | 98.97 | | 98.71 |
| *rpl22* | 85564 | 86031 | 468 | 97.01 | 96.58 | | 96.37 |
| *rpl23* | 153943 | 154224 | 282 | 99.29 | 99.29 | | 99.29 |
| *rpl32* | 114918 | 115082 | 165 | 95.76 | 96.97 | | 95.76 |
| *rpl33* | 69812 | 70012 | 201 | 99.00 | 99.50 | | 100.00 |
| *rpl36* | 81716 | 81829 | 114 | 95.61 | 98.25 | | 99.12 |
| *rpoA* | 80119 | 81132 | 1014 | 98.22 | 98.82 | | 98.03 |
| *rpoB* | 24146 | 27358 | 3213 | 98.91 | 99.28 | | 99.41 |
| *rpoC1* | 21337 | 22950 | 2067 | 99.01 | 98.82 | | 99.12 |
|  | 23688 | 24140 | - | - | - | | - |
| *rpoC2* | 17013 | 21179 | 4167 | 98.22 | 98.44 | | 98.56 |
| *rps11* | 81198 | 81614 | 417 | 98.56 | 99.76 | | 99.52 |
| *rps12* | 71915 | 72028 | 414 | 99.67 | 99.33 | | 99.33 |
|  | 141622 | 141921 | - | - | - | | - |
| *rps14* | 38410 | 38712 | 303 | 99.34 | 99.01 | | 99.01 |
| *rps15* | 125081 | 125344 | 264 | 97.73 | 97.35 | | 98.11 |
| *rps16* | 5075 | 5301 | 267 | 97.84 | 98.70 | | 99.54 |
|  | 6168 | 6207 | - | - | - | | - |
| *rps18* | 70199 | 70504 | 306 | 98.69 | 99.02 | | 98.69 |
| *rps19* | 86084 | 86362 | 279 | 98.92 | 98.92 | | 98.57 |
| *rps2* | 16082 | 16792 | 711 | 99.44 | 99.16 | | 99.16 |
| *rps3* | 84923 | 85579 | 657 | 97.72 | 98.93 | | 98.48 |
| *rps4* | 46935 | 47540 | 606 | 98.84 | 99.34 | | 99.34 |
| *rps7* | 142526 | 142993 | 468 | 100.00 | 100.00 | | 100.00 |
| *rps8* | 82276 | 82680 | 405 | 99.01 | 98.52 | | 98.77 |
| *ycf1* | 125741 | 131262 | 5522 | 93.09 | 93.55 | | 93.93 |
| *ycf15* | 146458 | 146742 | 285 | 99.62 | 100.00 | | 100.00 |
| *ycf2* | 146812 | 153615 | 6804 | 98.79 | 98.94 | | 98.98 |
| *ycf3* | 44079 | 44237 | 513 | 100.00 | 100.00 | | 100.00 |
|  | 44991 | 45220 | - | - | - | | - |
|  | 45961 | 46084 | - | - | - | | - |
| *ycf4* | 61193 | 62547 | 1355 | 98.56 | 99.10 | | 99.10 |
| *rrn16* | 138272 | 139761 | 1490 | 100.00 | 99.93 | | 99.33 |
| *rrn23* | 133267 | 136076 | 2810 | 99.82 | 99.79 | | 99.79 |
| *rrn4.5* | 133063 | 133165 | 103 | 100.00 | 100.00 | | 100.00 |
| *rrn5* | 109363 | 109483 | 121 | 100.00 | 100.00 | | 100.00 |

The percent identity of protein-coding sequences was calculated manually using ClustalW and NCBI BLAST 2 sequences programs.
